# Nutritional and Phytochemical Composition of Mediterranean Wild Vegetables after Culinary Treatment

**DOI:** 10.3390/foods9121761

**Published:** 2020-11-28

**Authors:** Patricia García-Herrera, Patricia Morales, Montaña Cámara, Virginia Fernández-Ruiz, Javier Tardío, María Cortes Sánchez-Mata

**Affiliations:** 1 Departamento Nutrición y Ciencia de los Alimentos, Facultad de Farmacia, Universidad Complutense de Madrid (UCM), Pza. Ramón y Cajal, s/n, E-28040 Madrid, Spain; patrigar@pdi.ucm.es (P.G.-H.); mcamara@ucm.es (M.C.); cortesm@ucm.es (M.C.S.-M.); 2 Instituto Madrileño de Investigación y Desarrollo Rural, Agrario y Alimentario (IMIDRA), Finca “El Encín”, Apartado 127, E-28800 Alcalá de Henares, Spain; javier.tardio@madrid.org

**Keywords:** underutilized greens, thermal treatment, nutritional composition, folates, vitamin C, minerals

## Abstract

Studies are scarce on the nutritional and phytochemical composition of wild edible Mediterranean plants after culinary processing. This work provides the nutritional composition after culinary treatment (including dietary fiber and mineral composition) and bioactive compounds (folates, vitamin C and organic acids) of wild *Rumex pulcher* L., *Silene vulgaris* (Moench) Garcke. leaves, and wild *Asparagus acutifolius* L., *Bryonia dioica* Jacq., *Humulus lupulus* L., *Tamus communis* L. young shoots. Shoots better preserved their nutrients than leaves, due to their different tissue structure. Fresh and cooked wild greens present high dietary fiber values, and remained at remarkable levels after boiling. Na, K, Mg and Zn were lost in about 50% due to culinary processing, while Ca, Cu, Fe and Mn were more stable. Boiled leaves of *S. vulgaris* remained as a good Mn source. A portion of 100 g of most of the cooked analyzed species could cover a relevant percentage of the daily requirement of folates (*R. pulcher* and *A. acutifolius* providing more than 80%) and vitamin C (*T. communis* and *A. acutifolius* providing more than 35%).

## 1. Introduction

The Mediterranean basin is characterized by an enormous biodiversity and a rich heritage of edible wild plants, which since Ancient times have represented an important source of nutrients for rural communities, both for food and medicinal uses. However, in the last century, the industrialization of agriculture, together with migratory phenomena, as well as other factors, have contributed to an important loss of the knowledge acquired over generations and has drastically changed the ways of life of rural communities regarding the use of these edible wild resources [1].

Despite this decline, several European countries, including Spain, still maintain the tradition of collecting and eating some wild plant resources, and nowadays, the growing demand for healthy foods and natural antioxidants—combined with the development of sustainable cuisine—have renewed interest in and promoted the culinary use of wild edible plants.

The implementation of traditional culinary knowledge has the potential to contribute to the diversification of income in depressed regions through the sale of high-quality local products, as it has been promoted in some community rural development policies [2,3]. The revitalization of some of these culinary traditions for their applications in haute-cuisine can also support this increased interest in these wild plant species [4]. In this sense, some wild species can be considered as a good material for food innovation due to their great versatility, as well as their great potential as a source of unusual colors and flavors.

Mediterranean wild vegetables are important sources of nutrients [5,6] and bioactive compounds [7,8,9,10,11], which are totally preserved when they are consumed in the fresh form. However, as with cultivated green vegetables [12,13,14,15], some species must be consumed after culinary treatments with different cooking techniques, such as boiling, frying, mixing with other ingredients in stews or other elaborations [16,17], as in the case of *Rumex pulcher* L., *Silene vulgaris* (Moench) Garcke. leaves, and *Asparagus acutifolius* L., *Brionya dioica* Jacq., *Humulus lupulus* L., *Tamus communis* L. young shoots, which could have an important effect on their nutritional composition. These processes are frequently necessary to improve their edibility and/or to remove undesirable compounds, such as oxalic acid; while in other cases, the process is part of the traditional behavior of its consumption. In black bryony (*T. communis*) and white bryony (*B. dioica*), some toxic compounds (saponins and triterpene glycosides, respectively), or some ribosome inactivating proteins, can be found in different parts of the plant, such as fruits and subterranean tubers, and the cooking process traditionally applied to the edible parts of these species may reduce those toxic substances, by either solubilization into cooking liquid or heat degradation [18,19,20,21,22].

Luckaj et al. [23] point out that, in the Mediterranean area, different preparation techniques were traditionally applied to wild edible plants. In Italy wild greens are usually eaten fried (often with eggs) after the initial boiling, or made into a soup. In southern France they are often eaten raw with dressing, whereas in Croatia most wild greens are boiled for a long time (usually nearly half an hour) and then dressed with olive oil. While in Spain the traditional cooking techniques were boiling and frying processes, with or without other ingredients, as previously stated by Tardio et al. [16,17].

However, as a consequence of these culinary processes, wild vegetables may lose thermolabile components, such as vitamin C or folates (vitamin B_9_), as well as mineral elements due to their dissolution in the liquid cooking medium [24,25]. Moreover, variations in the cooking conditions, such as maximum temperature reached, cooking time, pH, surface/volume relation, water used, oxygen and light presence, matrix and/or food type, as well as compounds sensitivity to these factors can influence the final composition [26,27]. Some research works have been conducted on the analysis of nutritional and phytochemical composition, as well as antioxidant activity of traditionally consumed wild edible plants all over the world [28,29,30,31,32,33,34,35,36,37,38]. For example, Trichopoulou et al. [28] reported data of the nutritional composition of Cretan pies elaborated with cultivated and wild vegetables (such as *Rumex* spp. and *Foeniculum vulgare* Mill) cooked with olive oil. Salvatore et al. [29] reported the boiling effect on antioxidants (carotenoids, phenolics, flavonoids, and ascorbic acid content), as well as the antioxidant capacity of selected wild edible greens traditionally consumed in southern Italy (*Asparagus acutifolius* L., *Borrago officinalis* L., *Cichorium Intybus* L., *Diplotaxis erucoides* L., *Sinapis Incana* L. and *Sinapus Nigra* L.). Amalraj and Pius [33] studied calcium content its absorption inhibitors in raw and cooked green leafy vegetables commonly consumed in India. Prasanna et al. [35], reported the cooking effect (boiling, steaming, and frying) on polyphenols, flavonoids, carotenoids and antioxidant activity of leaves of six edible plants locally collected in Sri Lanka. Sergio et al. [38] studied bioactive phenolics and antioxidant capacity of some wild edible Mediterranean greens affected by boiling, steaming and microwave-cooking treatments.

However, there are very few studies about the final composition of these wild plant foods after culinary processing. This is the first report about folate (vitamin B_9_) content in the cooked analyzed wild species.

Thus, the aim of the present study was to evaluate the cooking process effect in terms of nutritional and bioactive compounds of the selected Mediterranean wild edible greens (*Rumex pulcher* L., *Silene vulgaris* (Moench) Garcke., *Asparagus acutifolius* L., *Bryonia dioica* Jacq., *Humulus lupulus* L. and *Tamus communis* L.), in order to improve knowledge on these selected species, which are traditionally eaten after these types of culinary treatment in the Mediterranean areas.

## 2. Materials and Methods

### 2.1. Plant Material

Considering the high number of reports of wild vegetables being traditionally consumed after cooked vegetables in the Iberian Peninsula, [15,16,39], six wild edible greens were selected (Table 1). The samples included the basal leaves of *Rumex pulcher* L., the tender stems with leaves of *Silene vulgaris* (Moench) Garcke., and the young shoots of *Asparagus acutifolius* L., *Bryonia dioica* Jacq., *Humulus lupulus* L. and *Tamus communis* L.

As shown in Table 1, the samples were collected in different locations of Central Spain (Table 1). In order to obtain a representative sample, an amount of 500 g of the edible part of each species were selected from more than 25 plants randomly chosen in optimal conditions for consumption. The samples were collected in spring, when the edible parts were tender, which is the optimal moment for consumption. After gathering and preparation (cleaning, discarding altered parts), fresh plants were packed in plastic bags and transported to the laboratory in a cold system within the day. Then they were analyzed for moisture and vitamin C, as well as organic acids, while one portion was immediately freeze-dried and preserved in hermetic containers at −20 °C, in a dark and dry atmosphere, until used for the analysis of total available carbohydrates (TAC), dietary fiber, proteins, lipids, ashes, mineral elements (Na, K, Ca, Mg, Cu, Fe, Mn and Zn) and vitamin B_9_ (folates).

Another portion of the raw material was boiled using traditional conditions: the greens were cut and added to boiling water (200 g of fresh plant per litre of water) for 10 min at 100 °C. Once cooked, the plant material was filtered from the cooking liquid, weighted, and subjected to the same analysis as the raw vegetables.

### 2.2. Analytical Methods

#### 2.2.1. Moisture

Moisture content was determined by desiccation following the AOAC 984.25 method [40] to constant weight at 105 °C (oven Memert, Genesys, Daly City, CA, USA).

#### 2.2.2. Crude Protein

Total protein was determined as nitrogen content by the Kjeldahl method, after digestion with H_2_SO_4_ distillation over 0.1 N H_2_SO_4_ and titration against 0.1 N NaOH. Total nitrogen content was converted to protein content by using the conversion factor 6.25 [40].

#### 2.2.3. Total Fat

A Tecator Soxtec System HT 1043 Extraction Unit (Fisher-Scientific, Madrid, Spain) was used to extract a freeze-dried sample with petroleum ether; then the extract was dried at 105 °C and cooled and weighed following the AOAC 983.23 method [40].

#### 2.2.4. Total Available Carbohydrates (TAC)

This analysis was carried out by a colorimetric method using an anthrone reagent, as described by Osborne and Voogt [41]. The methodology was optimized by [5], and involved hydrolysis of freeze-dried sample with HClO_4_ for 18 h in the dark, filtration and adjustment to 250 mL; then anthrone solution in 70% (v/v) H_2_SO_4_ was added to extract. Samples were kept in a boiling water bath for 12 min and absorbance was measured at 630 nm on a UV/Vis Spectrometer EZ210 (Perkin Elmer, Waltham, MA, USA) equipped with Lambda software PESSW ver. 1.2. The absorbance of the sample solution was compared to a 10–100 mg/mL concentration range standard glucose calibration curve.

#### 2.2.5. Total Dietary Fiber Content

AOAC enzymatic–non-gravimetric methods (993.21) were used for total dietary fiber analysis [40]. Waste was dried at 100 °C, and ash and protein contents were determined in the residue.

#### 2.2.6. Ash Content and Mineral Composition

For ash determination, the method 930.05 of AOAC was used [40], as adapted by García-Herrera et al. [5]. Incineration under high pressure was performed in a microwave oven (Muffle Furnace mls1200, Thermo Scientific, Madrid, Spain) for 24 h at 550 °C, and ashes were gravimetrically quantified. The residue was extracted with HCl (50% v/v) and HNO_3_ (50% v/v) to measure Fe, Cu, Mn and Zn were directly quantified. To avoid interferences between different elements, a dilution with La_2_O_3_/HCl was performed to analyse Ca and Mg; and with CsCl to analyse Na and K. All measurements were performed in atomic absorption spectroscopy (AAS) in Analyst 200 Perkin Elmer equipment (Perkin Elmer, Waltham, MA, USA), comparing absorbance responses with N 99.9% purity analytical standard solutions for AAS made with Fe(NO_3_)_3_, Cu(NO_3_)_2_, Mn(NO_3_)_2_, Zn(NO_3_)_2_, NaCl, KCl, CaCO_3_ and Mg band, supplied by Merck (Darmstadt, Germany) and Panreac Química (Barcelona, Spain).

#### 2.2.7. Folic acid and Folates (Vitamin B_9_)

Given the complexity of the different forms of natural folates with vitamin B_9_ activity occurring in the plants, a method was previously optimized by Morales et al. [11]. It involved the derivatization of the different chemical forms into 5-CH_3_-H_4_folate (mono and diglutamate) and HPLC-FL quantification. Briefly, the sample was extracted with a phosphate buffer of 100 mM Na_2_HPO_4_ × 2 H_2_O, pH 7 at 80 °C during 15 min, and centrifuged at 7000 rpm during 15 min. An aliquot of 5 mL of the supernatant obtained was incubated for 3 h at 37 °C, under stirring with rat serum and pancreatic chicken solution (5 mg/mL in phosphate buffer 100 mM, pH 7), providing deconjugase (γ-glutamate pteroilpoli-hydrolase) to release folates from starch and proteins in the plant food matrix. Then, folates were reduced with NaBH_4_ into 5-CH_3_-H_4_folate and passed through SPE/SAX cartridges, previously activated. The total amount of folic acid (pteroylglutamic acid) and their polyglutamates in the samples were measured by HPLC-fluorescence, using a RP 18 endcapped Lichrospher 100 (Merck) 250 × 5 mm (5 μm), and a gradient of acetonitrile and 100 mM sodium phosphate buffer (pH 4.4) was used as reported by Morales et al. [11] at a flow-rate of 0.4 mL/min. Identification of chromatographic peaks was performed comparing retention times with those obtained from commercial pure standards of 5-CH_3_-H_4_folate monoglutamate and pteroyl diglutamic acid, subjected to the same derivatization as the samples. Quantification was based on the FL signal response, and the resultant peak areas in the chromatograms were plotted against concentrations obtained from standards. Total folates contents in non-cultivated greens are expressed in 5-CH_3_-H_4_folate (mono and diglutamate) in μg/100 g of fresh weight.

#### 2.2.8. Vitamin C and Organic Acids

Vitamin C (Ascorbic and dehydroascorbic acid), as well as individual organic acids (oxalic, glutamic, malic, and citric), was determined based on protocols described by Sánchez-Mata et al. [42], using an HPLC-UV methodology after samples extraction with 4.5% *m*-phosphoric acid. The HPLC equipment used was a liquid chromatograph (Micron Analítica, Madrid, Spain) equipped with a Sphereclone ODS (2) 250 × 4.60, 5 μm Phenomenex column, isocratic pump (model PU-II), an AS-1555 automatic injector (Jasco, Japan), and a UV-visible detector (Thermo Separation Specta Series UV100), working at 245 nm for AA or 215 nm for organic acids. The mobile phase was 1.8 mM H_2_SO_4_ (pH = 2.6), with a flow rate of 0.9 mL/min for AA or 0.4 mL/min for organic acids. The compounds were identified by chromatographic comparisons with authentic standards (AA, oxalic, malic, and citric acids, all from Sigma, St. Louis, MO, USA), and glutamic acid (Merck, Germany) using linear calibration curves of all compounds for quantification purposes. All data were analyzed using Biocrom 2000 3.0 software (Biocrom, Madrid, Spain). Vitamin C and organic acids content were expressed in mg/100 g of fresh weight (fw).

### 2.3. Statistical Analysis

Triplicate analysis was done for all the samples. In tables, mean values ± standard deviation (SD) of three replicates is given for all the measured parameters. Retention of nutrients through the processing of the samples was calculated as apparent retention, as defined by [43,44]. The data obtained in the present study were analyzed by means of Wilcoxon Student’s t test, using α = 0.05 as the level for statistical significance. Moreover, multivariate principal component analysis (PCA) was applied using Statgraphics Plus 5.1 software (Warrenton, VA, USA).

## 3. Results and Discussion

The proximate composition (moisture, TAC, dietary fiber, proteins, total fat and ashes), mineral contents, as well as vitamins B_9_ and C, and organic acids of the wild vegetables analyzed, raw and cooked, are presented in tables (Table 2, Table 3, Table 4, Table 5 and Table 6).

As it can be observed in Table 2, all the parameters were highly affected (*p* < 0.05) by cooking process in the analyzed samples, except for *B. dioica* young shoots, which experiment lower variations in macronutrients.

**Figure 1:**
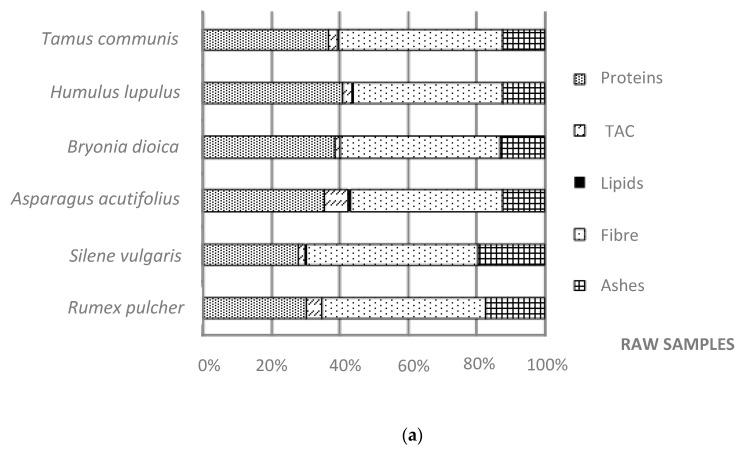

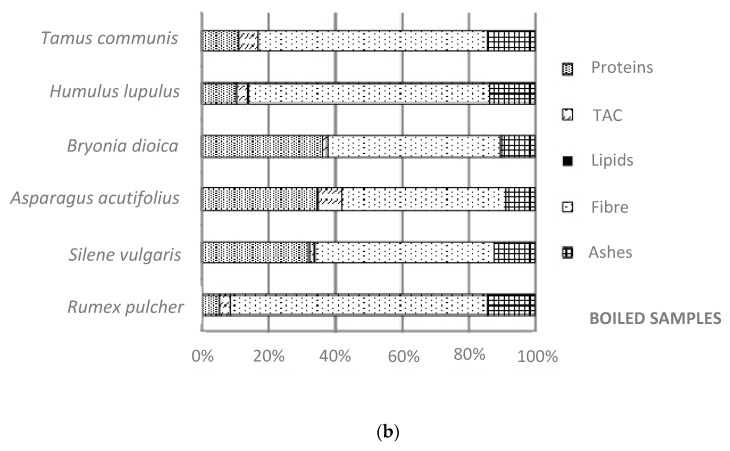
Proximate composition (**a**) before and (**b**) after boiling process in the wild edible greens selected (g/100 g dry basis).

As expected, moisture increased their content in cooked vegetables due to water absorption in all wild edible greens, being *S. vulgaris* tender leaves, the sample that absorbed a higher amount of water (6%) (Table 2). Figure 1 shows the dry weight data, allowing correction of the variations produced by the moisture and comparison of the macronutrients before and after cooking treatment. Proteins content decreased in all the cases, particularly, *R. pulcher, H. lupulus* and *T. communis* presented the highest losses with percentage percentages (89%, 87 and 83%, respectively), while *A. acutifolius* presented the best retention percentage (16% loss). Protein losses may be related to the denaturalization process due to heat treatment, which leads the smaller peptides and amino acids dissolution in the liquid medium.

Regarding available carbohydrates fraction, soluble sugars may also be dissolved into the cooking liquid while polymers may be thermally hydrolysed and then dissolved on it. *R. pulcher* and *S. vulgaris* tender leaves showed a relevant decrease in TAC (39 and 25% losses, respectively), while for other species, such as the young shoots of *B. dioica, A. acutifolius* and *H. lupulus* analyzed, this content was not significantly affected (*p* < 0.05) by cooking process. Even in some cases this fraction was slightly increased, attributable to some hydrolysis of the some fiber polymers, which may be converted in simple sugars, thus increasing the TAC fraction.

Moreover, after boiling processes, some losses in dietary fiber were also found in the studied species. However, when expressed as dry basis, dietary fiber content increased in all analyzed samples (Table 2), showing higher retention than any other nutrient. This observation is in agreement with the results of Amalraj and Pius [33] on Indian leafy wild vegetables. Based on EFSA recommendations (Dietary Reference Values published in 2017), it can be calculated that a portion of 100 g of these boiled wild plants, contributed by 11.6–17.6% to the daily requirements of dietary fiber, established as an Adequate Intake (AI) of 25 g/day for adults [45]).

It is clearly observed that all the species suffer an important loss in total ashes and therefore in their macro and microelements content due to the high-water solubility of minerals. However, young shoots presented higher ashes retention (34−44% losses) comparing with the tender-leaves species after the cooking process, as in the case of *R. pulcher* and *S. vulgaris* (with 47% and 65% of ashes losses, respectively) (Table 2).

However, macro and microelements showed different tendencies, due to the different forms in which they are present in the plant tissues. For example, K as free ions is widely distributed in food egetables, while others such as Fe can be found in this food matrix bonded to proteins or other high- molecular weight compounds [46].

The minerals that suffer the highest losses after the boiling process were Na, K, Mg and Zn. As it can be observed in Table 4, fresh *S. vulgaris* tender leaves stood out by its Mn content, with this content stable despite culinary treatment (0.6 mg Mn/100 g). When comparing with the nutritional recommendations of this mineral, it can be noted that 20% of the Mn adequate intake (AI) defined by [45] could be covered with the consumption of 100 g of this plant, even after boiling; accordingly, *S. vulgaris* can be highlighted as a good Mn source. Regarding Mg content, the losses were also higher in leaves than young shoots. Taking into account the Na content in analyzed wild greens, *R. pulcher* fresh leaves was the species with the highest Na content, however the cooking process highly promotes Na losses, which is especially relevant in this species (Table 3).

On the other hand, as expected, Ca, Cu and Fe were more stable, because these minerals can be linked to other molecules inhibiting their water solubilisation. This behavior is in agreement with other studies, reported by Amalraj and Pius [33], showing that calcium retention in wild vegetables is higher than other minerals; this can be related to some binding to other components in the food, such as oxalates, that inhibit extraction into the cooking liquid. *Bryonia dioica* and *Silene vulgaris* were the species that presented the higher Fe losses after boiling.

**Figure 2:**
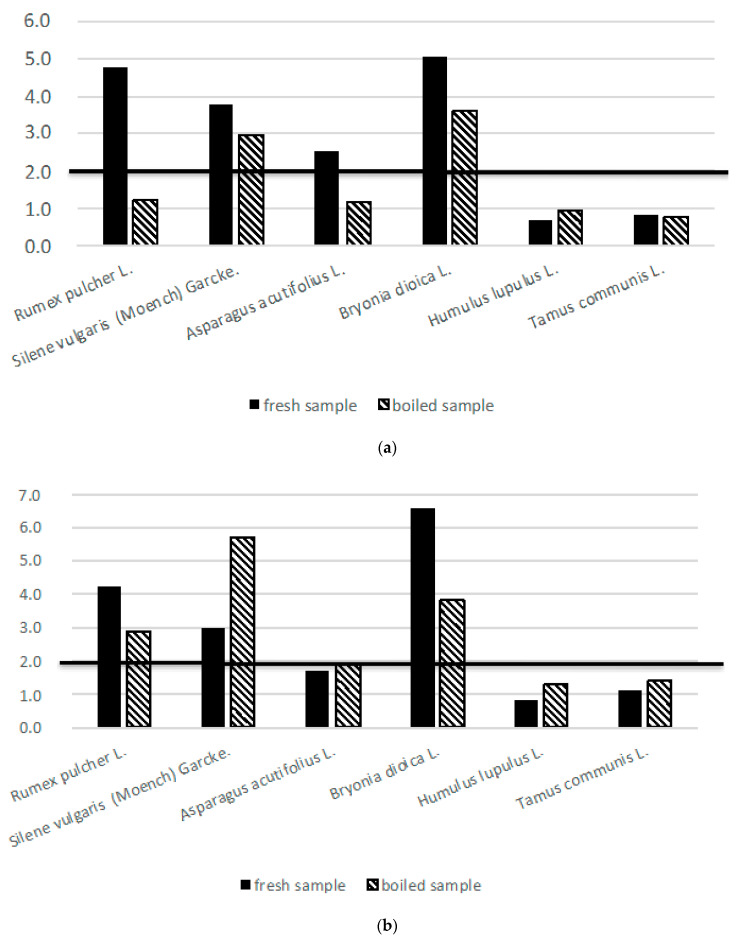
Variations detected after boiling process on: (**a**) oxalic acid/Ca molar ratio; (**b**) oxalic acid/Mg molar ratio.

Thus, the binding to other compounds is an important aspect that should be taken into account from a nutritional point of view. Foods may also contain anti-nutritional factors, which render some nutrients unavailable for absorption, with oxalic acid as the most important one in the case of green vegetables [47]. For that reason, the relation between calcium and oxalic acid content in the analyzed wild vegetables is discussed below (Figure 2).

It is well established that cultivated green vegetables are a really good source of folates (vitamin B_9_), in this way, Morales et al. [11] also reported wild edible vegetables as a relevant source of this vitamin. However, folates content after culinary processing in these wild edible greens have not been previously reported. Folates content stability during heat treatment depends, among other factors, on the relation surface/volume of food, the presence of some cations such as Fe, or nitrates presence, while the presence of antioxidants (such as vitamin C, phenolic compounds, etc.) favors a higher retention of folates during the culinary process [48]. The analyzed fresh samples (Table 5) are highlighted due to the relevant amount of this vitamin, particularly in the case of *R. pulcher* (0.397 mg/100 g), *S. vulgaris* (0.519 mg/100 g) and *A. acutifolius* (0.589 mg/100 g), as well as in some cooked samples, such as *R. pulcher* (80.277 mg/100 g) and *A. acutifolius* (0.283 mg/100 g). In all cases, the consumption of 100 g of most of these species (fresh and/or cooked) could cover more than 100% of the daily amount of folates needed, established as a population reference intake (PRI) of 330 μg/day for adults ≥18 years. Moreover, the previously mentioned fresh species could cover around 86–98% of the PRI for pregnant women (600 μg/day), and boiled *R. pulcher* leaves and *A. acutifolius* shoots could cover 47.2 and 46.2% of the PRI for the same population group.

As it can be seen in Table 5, a significant reduction of folates content was observed after processing in all the analyzed species. The lower retention was observed in *S. vulgaris* (19.6%), reaching a final amount of 0.102 mg/100 g in the cooked vegetable. Although it can be thought that leafy vegetables lose nutrients more easily due to the liquid cooking medium, compared to stems and shoots, folates do not show this behavior, as previously reported by Lin and Lin [49]. In agreement with this, the present work shows that the leaves of *R. pulcher* display a better retention of folates (69.78%) than other samples, with 0.277 mg/100 g in the cooked sample. Additionally, it should be noted that the wild green, *A. acutifolius* shoot, has the highest folate content from those analyzed in the raw and cooked samples (0.587 and 0.280 mg/100 g, respectively).

As a result of this study, it can be stated that the analyzed wild vegetables are good sources of folates even after traditional cooking process (Table 5), since their final contents still represent more than 30% of dietary recommendations [45].

Regarding vitamin C, as expected, the studied wild greens significantly reduced their vitamin C contents after cooking. As it is well known, this vitamin presents a high thermal instability. As it can be observed in Table 5, *R. pulcher, A. acutifolius, T. communis* and *H. lupulus*, presented high vitamin C contents in the raw material (46.5, 56.7, 58.6 and 60.9 mg/100 g fw, respectively), with *A. acutifolius* and *T. communis* being the samples with the highest vitamin C retention percentages after processing (61.9% and 86.7%, respectively). Lower ascorbic acid and total vitamin C amounts were reported by Salvatore et al. [29] in selected wild greens boiled during 15 min.

Taking into account that the intake of vitamin C (PRI for adults ≥18 years, published by [45]) should be at least of 95 mg/day for women and 110 mg/day for men, even considering the most exigent value (110 mg/day), a portion of 100 g of all the fresh analyzed wild greens could provide at least the 20% of PRI for adults; and those with better retention of vitamin C (*T. communis* and *A. acutifolius)* still cover 37% and 43% of PRI for adults with a 100 g of cooked portion, a relevant contribution to daily intake. On the other hand, *S. vulgaris* and *B. dioica* drastically reduced their contents to only 13.4 and 6.4 mg/100 g, respectively.

It can also be stated from the present research that AA was generally more stable than DHAA, and that the high vitamin C contents may be related to a higher retention of folates, as previously stated by other authors [48]. For example, *A. acutifolius* was the species with the higher retention of folates and vitamin C together.

Many organic acids are involved in ascorbic acid metabolism in plants [50], and different authors have suggested their role in the reactions of the oxidation-reduction of tocopherols [51,52]. For that reason, the knowledge of their content in plant tissues, as well as their behavior through the cooking process, has been considered as interesting information that can help to explain the relationships between antioxidant compounds presence in the vegetables (namely ascorbic acid) and vitamin stability (as in the case of folates).

Table 6 shows the organic acid composition of the analyzed samples. Glutamic acid was only found in *A. acutifolius* and *B. dioica* young shoots, with losses of 69% and 100% respectively, after the culinary process. Malic acid was identified in the tender shoots of *A. acutifolius, B. dioica* and *T. communis*, with *B. dioica* having the highest content in either raw or cooked material and *T. communis* having the highest retention percentage. Citric acid was relatively stable in almost all the wild greens studied, with the exception of *R. pulcher* tender leaves with 33.6% retention, while *T. communis* had the most presence of this organic acid.

However, oxalic acid was the most relevant organic acid found in the analyzed samples, due to its role as an antinutrient. This compound, when ingested in high amounts, is excreted in urine, causing hyperoxaluria, which leads to the deposition of calcium oxalate in kidney tissue or crystallization as calcium oxalate kidney calculus (nephrolithiasis). The minimal lethal dose of this organic acid for adults has been established as 5 g per day, which is a very difficult level to reach in a normal and balanced diet. However, people with a sensitivity to form this kind of calculus should reduce the amount of oxalic acid in the diet to avoid this problem. Besides this, oxalate forms insoluble complexes with minerals such as Ca, and to a lesser extent Mg, in the gut; the insoluble complexes precipitate and are not absorbed, leading to a lower bioavailability of these minerals, and also less absorption of oxalates. Thus, a high intake of these minerals is desirable to impair the negative effects of oxalic acid, as both the urinary deposition of absorbed oxalic acid, and the reduction of minerals absorption [47,53,54,55].

The results in Table 6 show that the studied vegetables have oxalic acid contents from 80 to 730 mg/100 g, which are high amounts; however, with the exception of *S. vulgaris*, generally, those plants with higher oxalic acid amounts in the raw material (*B. dioica* and *R. pulcher*) lose this compound in a higher extent after culinary treatment (retentions of 36 and 39% respectively), while those with lower amounts (*A. acutifolius, H. lupulus* and *T. communis*) showed higher oxalic acid retentions (82%). From this, the removal of cooking liquids in oxalate-rich vegetables is desirable for people with the ability of easily form this oxalate kidney calculus, and also to avoid a decrease in dietary calcium bioavailability. Additionally, it can be stated that boiled *Asparagus acutifolius, Bryonia dioica, Humulus lupulus* and *Tamus communis* presented lower levels of oxalic acid, and thus they can be a better choice for this group of population.

As previously mentioned, the effects of oxalic acid in the human body are conditioned by the presence of cations in foods. For that reason, the ratios of oxalic acid to minerals have been proposed as indicators of the nutritional quality of foods: a high ratio would mean high oxalates absorption (with the risk of urinary calculus) and impaired mineral absorption; a low ratio would mean more minerals available to be absorbed and a higher capacity to reduce oxalates absorption and urinary deposition.

In this study, the molar ratios of oxalic acid/Ca and oxalic acid/Mg have been calculated for all the wild vegetables studied, either in raw and cooked material, to establish the effect of the cooking process on both the availability of minerals and negative effects of food oxalates. In accordance with Israr et al. [54], the molar ratio of oxalic acid to Ca or Mg above 2 would mean an excess of oxalic acid and impaired mineral absorption, while a value below 1 would minimize this negative effect. As it can be seen, from the studied samples, *H. lupulus* and *T. communis* are always below an oxalic acid/Ca ratio of 1; accordingly, they would be better options for people with a low calcium intake in their diet. Other species such as *R. pulcher* and *A. acutifolius* present high oxalic acid/Ca ratios, but this ratio notably decreases after boiling, which means that thermal treatment helps to increase the amount of available calcium in this species. On the contrary, *B. dioica* or *S. vulgaris*, in both raw and boiled forms, always present high values for the oxalic acid/Ca ratio (>2). Other authors also reported that the total oxalate level in plant foods was inversely correlated with the calcium bioavailability [33,56]. For this reason, a thorough cooking process has been recommended for oxalate-rich food to induce some dissolution of soluble oxalates into the cooking liquid.

Regarding oxalic acid/Mg ratio, similar behavior was found (Figure 2), with the exception that in some cases this ratio slightly increases through the cooking process, with can be attributed to the higher solubility of magnesium salts than oxalic acid into the cooking liquid; Israr et al. [54] also reported the higher solubility of magnesium oxalates than calcium oxalates in foods. Despite the slight increase, *H. lupulus* and *T. communis* shoots remain as species with more adequate oxalic acid/Mg ratios (not higher than 1,4), before and after boiling; *A. acutifolius* showed a ratio close to 2, which is still adequate; on the contrary, *R. pulcher, B. dioica* and *Silene vulgaris* presented a too high molar ratio oxalic acid/Mg.

For this reason, from the point of view of Ca and Mg availability and for people suffering of kidney calculus, the boiled shoots of wild *H. lupulus* and *T. communis*, and to a lesser extent *A. acutifolius*, are better choices; while *B. dioica* shoots and *R. pulcher*, and *S. vulgaris* leaves should be avoided.

**Figure 3:**
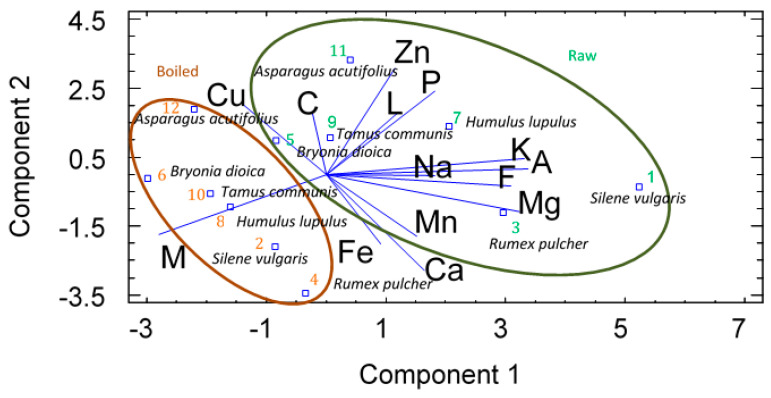
Principal component analysis (PCA) graphs of nutrients from the raw (1, 3, 5, 7, 9 and 11) and boiled vegetables (2, 4, 6, 8, 10 and 12) investigated. Numbers on the plot represent analyzed vegetables. Sample numbering for raw and boiled respectively: 1, 2: *Silene vulgaris*; 3, 4: *Rumex pulcher*; 5, 6: *Bryonia dioica*; 7, 8: *Humulus lupulus*; 9, 10: *Tamus communis*; 11, 12: *Asparagus acutifolius* L.: lipids; P: proteins; C: carbohydrates; F: fiber; A: ashes; M: moisture.

Finally, multivariate analysis, a principal component analysis (PCA), which reduces the multidimensional structure of the data and provides a two-dimensional map explaining the variance observed, was carried out (Figure 3). This figure shows the difference between various wild vegetables analyzed, raw and cooked, using principal component analysis (PCA) based on the profiles of moisture, TAC, dietary fiber, proteins, total fat, ashes and mineral contents. It can be observed in Figure 3 that the obvious differences in the profiles of particular vegetables species, raw and cooked, vary substantially in terms of their moisture content. There are particular meaningful differences between some of the investigated vegetables, which are especially noteworthy for raw *Rumex pulcher* and *Asparagus acutifolius*. Examination of the PCA graphs shows a similarity between *Bryonia dioica* and *Tamus communis*, and also between *Silene vulgaris* and *Rumex pulcher* compounds in raw vegetables. When the data are compared for cooked vegetables, the similarities between *Bryonia dioica* and *Tamus communis* can still be observed (close distance between clusters), although the other vegetables species have smaller distance between clusters. Remarkably, after cooking, the differences between wild vegetables are less pronounced.

The first and second component explained 64.61% of the total variability in the composition of wild vegetables investigated. The first principal component (40.24% of the total variance) was highly correlated with dietary fiber, mineral content, K, Mg and in lesser degree with Na, while it was negatively correlated with moisture content. The second principal component (24.37% of the total variance) was highly correlated with protein, Zn, Cu and in minor degree with carbohydrates and lipids while it was negatively correlated with Fe and Ca content.

Raw *Silene vulgaris*, *Rumex pulcher* and *Humulus lupulus* were mostly characterized by the first component with high fiber, K, Mg content and low moisture content, while *Bryonia dioica* was negatively characterized by these compounds. Moreover, *Tamus communis* and *Asparagus acutifolius* could be considered rich in protein Zn, Cu and to a lesser extent in carbohydrates content characteristics related to component 2.

Raw wild vegetables analyzed were in the first, second and third quarter. However, those boiled were represented in the first and fourth quarter of the biplot. This data representation showed that the culinary technic affected in a great manner the mineral composition of the vegetables more than the proximate composition; this can be explained by the fact that ash and many minerals were negatively correlated with moisture content.

## 4. Conclusions

As expected, the boiling process supposes an increase of water content in all the vegetables studied, as well as protein and mineral content reduction. Dietary fiber stands as a major compound also in the cooked wild greens studied, providing 9–17% of daily recommendations (in a 100 g consumed portion). Na, K, Mg and Zn contents in the cooked vegetables are lost in approximately 50%. On the contrary, Ca, Cu, Fe and Mn remain more stable. The cooked tender leaves of *Silene vulgaris* are still a good Mn source (0.6 mg/100 g). Regarding vitamin retention, most of the analyzed species, even after boiling, could cover a relevant percentage of the PRI of folates and vitamin C; some species deserve a especial mention for remaining as very good sources of some vitamins, even after boiling, such as *R. pulcher* as a source of folates, *T. communis* as a source of vitamin C and *A. acutifolius* as a source of both vitamins.

Moreover, the boiling process can reduce the amount of the antinutrient oxalic acid in wild greens. It can be especially interesting in the case of the leaves of *R. pulcher*, reducing oxalic acid/Ca ratio through the loss oxalic acid, while Ca is kept in considerable amounts, thus improving calcium availability. This species, as well as the young shoots of *A. acutifolius, H. lupulus* and *T. communis*, after boiling, are good options from the point of view of oxalic acid/Ca ratio.

From all these results, the knowledge of the nutrient composition of some traditionally eaten wild greens is achieved, and its role as a source of nutrients even after culinary processing has been demonstrated.

## Figures and Tables

**Table 1:** Description and location of the sampled wild vegetable species.

Family	Species	Edible Part	Site	
*Polygonaceae*	*Rumex pulcher* L.	Basal leaves	Alcalá de Henares	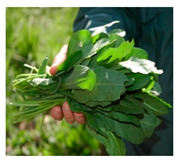
*Caryophyllaceae*	*Silene vulgaris* (Moench) Garcke.	Tender stems with leaves	Cadalso de los Vidrios	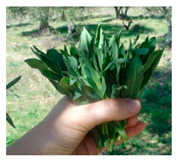
*Asparagaceae*	*Asparagus acutifolius* L.	Young shoots with or without leaves	Alcalá de Henares	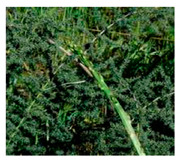
*Cannabaceae*	*Humulus lupulus* L.	Young shoots with or without leaves	Alcalá de Henares	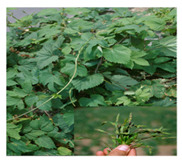
*Cucurbitaceae*	*Bryonia dioica* Jacq.	Young shoots with or without leaves	Alcalá de Henares	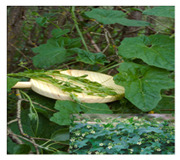
*Dioscoreaceae*	*Tamus communis* L.	Young shoots with or without leaves	Tres Cantos	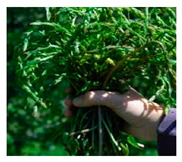

Photographs (J. Tardio).

**Table 2:** The proximate composition of raw and boiled wild vegetables (g/100 g fresh weight).

Species		Moisture	Proteins	Lipids	TAC	Dietary Fiber	Ashes
*Rumex pulcher* L.	Fresh	88.1 ± 0.1 *	2.70 ± 0.01 *	0.10 ± 0.02 *	3.31 ± 0.10 *	4.23 ± 0.03 *	1.54 ± 0.02 *
	Boiled	90.8 ± 0.9	0.31 ± 0.02	0.03 ± 0.00	2.02 ± 0.12	4.41 ± 0.07	0.81 ± 0.01
*Silene vulgaris* (Moench) Garcke.	Raw	86.1 ± 0.1 *	3.16 ± 0.09 *	0.33 ± 0.02 *	1.50 ± 0.07 *	5.64 ± 0.06 *	2.18 ± 0.03 *
	Boiled	92.1 ± 0.4	1.98 ± 0.02	0.22 ± 0.02	1.13 ± 0.03	3.28 ± 0.03	0.77 ± 0.02
*Asparagus acutifolius* L.	Raw	86.9 ± 0.1 *	3.00 ± 0.13 *	0.44 ± 0.02 *	4.46 ± 0.19	3.73 ± 0.06 *	1.00 ± 0.04 *
	Boiled	89.6 ± 0.1	2.52 ± 0.02	0.12 ± 0.00	4.99 ± 0.45	3.56 ± 0.02	0.57 ± 0.03
*Bryonia dioica* L.	Raw	90.7 ± 0.1 *	3.05 ± 0.21	0.11 ± 0.01	1.06 ± 0.07	3.69 ± 0.05 *	1.03 ± 0.03 *
	Boiled	93.4 ± 0.2	2.01 ± 0.44	0.15 ± 0.02	1.21 ± 0.18	2.88 ± 0.03	0.62 ± 0.02
*Humulus lupulus* L.	Raw	86.2 ± 0.1 *	4.23 ± 0.49 *	0.10 ± 0.01 *	2.02 ± 0.21	4.50 ± 0.15 *	1.26 ± 0.01 *
	Boiled	90.7 ± 0.2	0.56 ± 0.01	0.15 ± 0.01	1.89 ± 0.17	3.81 ± 0.03	0.72 ± 0.01
*Tamus communis* L.	Raw	88.7 ± 0.4 *	3.14 ± 0.06 *	0.20 ± 0.01 *	2.02 ± 0.21 *	4.06 ± 0.10 *	1.03 ± 0.01 *
	Boiled	90.6 ± 0.1	0.55 ± 0.05	0.08 ± 0.00	3.07 ± 0.19	3.33 ± 0.04	0.68 ± 0.03

TAC: Total available carbohydrates. Data expressed as mean ± standard deviation (*n* − 1). *n* = 3. * Statistically significant differences (*p* < 0.05), between raw and boiled samples.

**Table 3:** Mineral macro-elements in raw and boiled wild vegetables (mg/100 g fresh weight).

Species		K (mg/100 g)	Na (mg/100 g)	Ca (mg/100 g)	Mg (mg/100 g)
*Rumex pulcher* L.	Raw	520.8 ± 41.4 *	123.5 ± 9.4 *	68.2 ± 2.8 *	45.7 ± 1.0 *
	Boiled	206.1 ± 20.3	29.1 ± 0.2	107.1 ± 9.7	26.9 ± 1.8
*Silene vulgaris* (Moench) Garcke.	Raw	670.7 ± 69.0 *	21.8 ± 1.6 *	74.0 ± 3.3	56.0 ± 0.6 *
	Boiled	226.4 ± 3.7	11.8 ± 2.5	67.9 ± 5.4	21.1 ± 0.2
*Asparagus acutifolius* L.	Raw	338.1 ± 21.5 *	37.5 ± 3.0 *	17.6 ± 1.5 *	15.6 ± 0.9 *
	Boiled	177.1 ± 18.0	21.0 ± 1.9	31.5 ± 0.3	10.9 ± 1.8
*Bryonia dioica* L.	Raw	351.1 ± 12.9 *	21.9 ± 2.3	31.7 ± 1.2 *	14.6 ± 0.8 *
	Boiled	171.0 ± 10.2	18.1 ± 0.2	16.0 ± 1.5	9.0 ± 1.7
*Humulus lupulus* L.	Raw	421.3 ± 14.2 *	57.7 ± 8.1 *	58.3 ± 0.8 *	28.9 ± 1.0 *
	Boiled	171.8 ± 15.7	7.1 ± 0.7	40.4 ± 5.6	17.0 ± 0.6
*Tamus communis* L.	Raw	307.4 ± 9.4 *	16.3 ± 3.4	42.1 ± 2.7	19.0 ± 1.4
	Boiled	202.4 ± 22.9	14.9 ± 1.7	47.3 ± 3.3	15.2 ± 1.2

Data expressed as mean ± standard deviation (*n* − 1). *n* = 3. Different letters mean statistically significant differences (*p* < 0.05). * Statistically significant differences (*p* < 0.05), between raw and boiled samples.

**Table 4:** Mineral microelements in raw and boiled wild vegetables (mg/100 g fresh weight).

Species		Cu (mg/100 g)	Fe (mg/100 g)	Mn (mg/100 g)	Zn (mg/100 g)
*Rumex pulcher* L.	Raw	0.10 ± 0.01	1.26 ± 0.03	0.22 ± 0.01	0.39 ± 0.02 *
	Boiled	0.08 ± 0.01	1.10 ± 0.18	0.20 ± 0.02	0.18 ± 0.03
*Silene vulgaris* (Moench) Garcke.	Raw	0.01 ± 0.002	0.21 ± 0.00 *	0.55 ± 0.01 *	0.51 ± 0.01 *
	Boiled	0.06 ± 0.02	0.47 ± 0.02	0.60 ± 0.01	0.20 ± 0.01
*Asparagus acutifolius* L.	Raw	0.13 ± 0.01 *	0.36 ± 0.03	0.07 ± 0.003 *	0.85 ± 0.01 *
	Boiled	0.38 ± 0.08	0.22 ± 0.09	0.12 ± 0.01	0.42 ± 0.00
*Bryonia dioica* L.	Raw	0.26 ± 0.02 *	0.41 ± 0.02 *	0.13 ± 0.005 *	0.61 ± 0.03 *
	Boiled	0.10 ± 0.01	0.20 ± 0.005	0.08 ± 0.001	0.30 ± 0.03
*Humulus lupulus* L.	Raw	0.14 ± 0.02 *	0.58 ± 0.03	0.10 ± 0.002 *	0.82 ± 0.01 *
	Boiled	0.08 ± 0.001	0.57 ± 0.07	0.18 ± 0.02	0.40 ± 0.02
*Tamus communis* L.	Raw	0.14 ± 0.01 *	0.48 ± 0.02	0.11 ± 0.03	0.64 ± 0.01 *
	Boiled	0.09 ± 0.02	0.43 ± 0.04	0.13 ± 0.01	0.44 ± 0.04

Data expressed as mean ± standard deviation (*n* − 1). *n* = 3. Different letters mean statistically significant differences (*p* < 0.05). * Statistically significant differences (*p* < 0.05), between raw and boiled samples.

**Table 5:** Vitamins B_9_ (folates) and C in raw and boiled wild vegetables (mg/100 g fresh weight).

Species		Total Folates (mg/100 g)	% PRI	AA (mg/100 g)	DHAA (mg/100 g)	Total Vitamin C (mg/100 g)	% PRI
*Rumex pulcher* L.	Raw	0.397 ± 0.023 *	120.3	30.8 ± 0.7 *	16.1 ± 0.2 *	46.5 ± 0.1 *	42.3
	Boiled	0.277 ± 0.026	83.9	20.4 ± 0.7	1.2 ± 0.3	21.6 ± 0.9	19.6
*Silene vulgaris* (Moench) Garcke.	Raw	0.519 ± 0.140 *	157.3	14.3 ± 1.0	11.1 ± 0.9 *	25.1 ± 0.5 *	22.8
	Boiled	0.102 ± 0.012	30.9	13.9 ± 1.0	nd	13.4 ± 0.8	12.2
*Asparagus acutifolius* L.	Raw	0.589 ± 0.017 *	178.5	29.3 ± 0.1 *	26.0 ± 0.6	56.7 ± 0.6 *	51.5
	Boiled	0.283 ± 0.007	85.7	24.2 ± 0.5	23.6 ± 1.4	47.9 ± 1.8	43.5
*Bryonia dioica* L.	Raw	0.177 ± 0.008 *	53.6	12.0 ± 0.3 *	13.7 ± 1.7 *	25.7 ± 2.0 *	23.4
	Boiled	0.122 ± 0.015	36.9	6.4 ± 0.3	0.2 ± 0.1	6.4 ± 0.2	5.8
*Humulus lupulus* L.	Raw	0.304 ± 0.045 *	92.1	37.2 ± 0.2 *	23.2 ± 0.8 *	60.9 ± 0.1 *	55.4
	Boiled	0.177 ± 0.038	53.6	11.3 ± 0.5	5.6 ± 0.2	17.4 ± 0.8	15.8
*Tamus communis* L.	Raw	0.159 ± 0.004 *	48.2	56.9 ± 0.2 *	1.6 ± 0.1 *	58.6 ± 0.4 *	53.3
	Boiled	0.109 ± 0.022	33.0	36.2 ± 0.8	nd	40.9 ± 1.6	37.2

AA = ascorbic acid; DHAA = dehydroascorbic acid. nd = non detected; PRI = population reference intake. Data expressed as mean ± standard deviation (*n* − 1). *n* = 3. * Statistically significant differences (*p* < 0.05), between raw and boiled samples.

**Table 6:** Organic acids in raw and boiled wild vegetables (g/100 g fresh weight).

Species		Oxalic Acid	Glutamic Acid	Malic Acid	Citric Acid
*Rumex pulcher* L.	Raw	0.73 ± 0.02 *	nd	nd	0.102 ± 0.003 *
	Boiled	0.29 ± 0.02	nd	nd	0.034 ± 0.003
*Silene vulgaris* (Moench) Garcke.	Raw	0.63 ± 0.07 *	nd	nd	0.12 ± 0.01 *
	Boiled	0.45 ± 0.03	nd	nd	0.06 ± 0.02
*Asparagus acutifolius* L.	Raw	0.10 ± 0.01	0.042 ± 0.005 *	0.22 ± 0.04 *	0.33 ± 0.05
	Boiled	0.08 ± 0.00	0.029 ± 0.002	0.09 ± 0.01	0.20 ± 0.07
*Bryonia dioica* L.	Raw	0.36 ± 0.02 *	0.119 ± 0.004 *	1.71 ± 0.04 *	0.06 ± 0.01 *
	Boiled	0.13 ± 0.01	nd	0.98 ± 0.07	0.041 ± 0.005
*Humulus lupulus* L.	Raw	0.09 ± 0.02	nd	0.77 ± 0.17 *	0.17 ± 0.01
	Boiled	0.083 ± 0.007	nd	0.47 ± 0.02	0.12 ± 0.03
*Tamus communis* L.	Raw	0.08 ± 0.01	nd	nd	0.34 ± 0.02 *
	Boiled	0.08 ± 0.01	nd	nd	0.24 ± 0.01

nd = non detected; Data expressed as mean ± standard deviation (*n* − 1). *n* = 3. * Statistically significant differences (*p* < 0.05), between raw and boiled samples.
